# Dr. Frank H. Moyer

**Published:** 1907-10

**Authors:** 


Dr. Frank H. Moyer, of Moscow, N. Y., died at his home August 28, 1907, after a prolonged period of illness due to paralysis, aged 60 years. He graduated at the medical department, University of Buffalo, in 1872, and practised his profession in Moscow from that date until disabled by disease. He was a member of the Medical Society of the State of New York and of that of Livingston County and was one time president of the alumni association of Buffalo University medical college. He also served for several years as a member of the board of curators of his alma mater.

